# The role of structural variations in Alzheimer’s disease and other neurodegenerative diseases

**DOI:** 10.3389/fnagi.2022.1073905

**Published:** 2023-02-08

**Authors:** Hui Wang, Li-San Wang, Gerard Schellenberg, Wan-Ping Lee

**Affiliations:** ^1^Department of Pathology and Laboratory Medicine, Perelman School of Medicine, University of Pennsylvania, Philadelphia, PA, United States; ^2^Penn Neurodegeneration Genomics Center, Perelman School of Medicine, University of Pennsylvania, Philadelphia, PA, United States

**Keywords:** structural variations, Alzheimer’s disease, next-generation sequencing, short-tandem repeats, transposable elements, copy number variations

## Abstract

Dozens of single nucleotide polymorphisms (SNPs) related to Alzheimer’s disease (AD) have been discovered by large scale genome-wide association studies (GWASs). However, only a small portion of the genetic component of AD can be explained by SNPs observed from GWAS. Structural variation (SV) can be a major contributor to the missing heritability of AD; while SV in AD remains largely unexplored as the accurate detection of SVs from the widely used array-based and short-read technology are still far from perfect. Here, we briefly summarized the strengths and weaknesses of available SV detection methods. We reviewed the current landscape of SV analysis in AD and SVs that have been found associated with AD. Particularly, the importance of currently less explored SVs, including insertions, inversions, short tandem repeats, and transposable elements in neurodegenerative diseases were highlighted.

## Introduction

What is the genetic cause of Alzheimer’s disease (AD)? The answer to this question has not changed much for the past decade. 10–20% of early-onset familial forms of AD are caused by mutations in *APP*, *PSEN1,* and *PSEN2* (Tanzi et al., 1987; Levy-Lahad et al., 1995; Sherrington et al., 1995). Genome-wide association studies (GWASs) have confirmed the role of *APOE* alleles in late-onset AD (LOAD) and identified dozens of other variants with small effects. A most recent GWAS revealed 42 new risk loci associated with AD with the odd ratio between 0.89 and 1.47 (Bellenguez et al., 2022). Common single nucleotide polymorphisms (SNPs) altogether were estimated to explain about 30% of phenotypic variance for AD (Lee et al., 2013; Ridge et al., 2013), which were calculated to have a heritability about 70% (Gatz et al., 2006). Epistasis and structural variants (SVs) were expected to account for the missing heredity in AD (Bertram et al., 2010; Raghavan and Tosto, 2017). However, there was only a limited increase in predicting the status of AD when epistasis was incorporated into polygenic risk (Wang et al., 2020, 2021). Compared to SNPs, SVs are estimated to contribute equally or more to genetic variations by total nucleotide content (Feuk et al., 2006). From the previous study, SVs account for 17.2% of strongly deleterious rare variants in the human genome (Abel et al., 2020). Though SVs were less studied in the etiology of diseases due to challenges in characterizing SVs accurately and exhaustively from array and short-read data, they are likely to be an important component of the missing heritability in AD.

**Figure 1:**
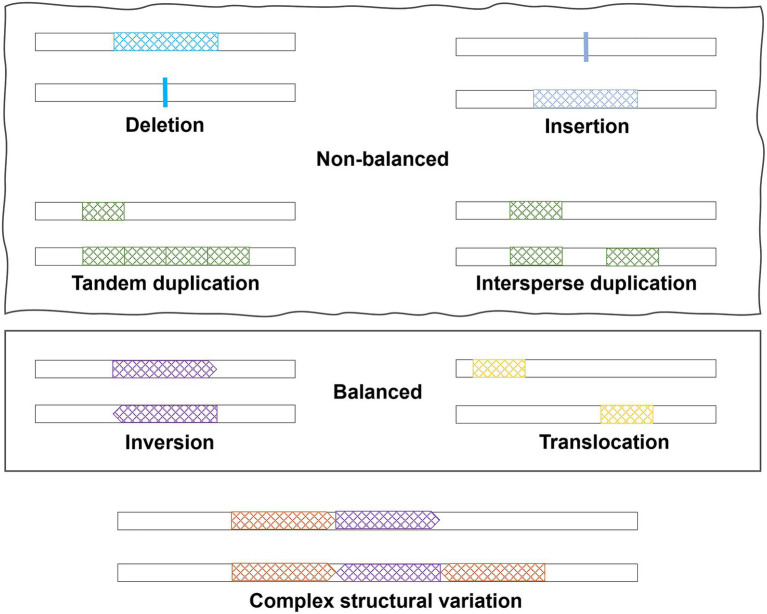
Different types of structural variations.

SVs are large genomic variations (>50 bp), including copy number variations (CNVs, i.e., deletions and duplications), insertions, inversions, translocations, and complex combinations (Figure 1). Deletions, duplications, and insertions are called non-balanced SVs as they increase or decrease the dosage of specific genes, while inversions and translocations are balanced SVs. Compared to SNPs, SVs may have a larger impact on the human genome and can alter not only protein coding sequences but also the dosage of a specific exon or entire gene. Considering the numerous publications of GWAS for SNPs, SVs in human diseases have been understudied due to limitations on SV detection methods. In this review, we started with a brief introduction of technologies and methods, which are commonly used to detect SVs. Subsequently, a summary of the current understandings of SVs in AD pathogenesis was provided. The review focused on the important roles of specific types of SVs in AD and neurodegenerative diseases and how recent sequencing technologies may help bring new insights.

## Genome-wide detection of structural variants

### Array-based methods

Large SVs at cytogenetic level (~2–3 Mb or above) can be identified by karyotyping, such as chromosomal aneuploidies in Down’s Syndrome (Jacobs et al., 1959). Later, fluorescence *in situ* hybridization (FISH) (Pinkel et al., 1986) allows detection of smaller deletions and duplications as well as translocations using fluorescent probes that only bind to specific genes or chromosome regions. Based on FISH, comparative genomic hybridization (CGH; Kallioniemi et al., 1992) was developed to produce a map of DNA sequence copy number throughout the whole genome. A broad application of CGH as a diagnosis tool requires higher resolution and simpler procedures, which leads to development of microarray-based comparative genomic hybridization (aCGH; Solinas-Toldo et al., 1997; Vissers et al., 2003; Bejjani and Shaffer, 2006) for a finer analysis of genomic CNVs. Since 2010, aCGH has been the first-tier cytogenetic test in place of G-banded karyotyping for patients with unexplained developmental delay or intellectual disability, autism spectrum disorders, and congenital anomalies (Manning et al., 2010; Miller et al., 2010). In aCGH, array can be built with DNA sequences whether from oligonucleotides, cDNAs, or bacterial artificial chromosomes. Then, reference and test DNA labeled with two different fluorophores probes are hybridized to the DNA sequences on the array (Bejjani and Shaffer, 2006). Comparing the fluorescence intensity between case and control, genomic gains or losses can be attained simultaneously. The disadvantage of aCGH is that it cannot provide the absolute copy number of a specific gene or region.

As the wide application of GWAS, numerous algorithms, such as PennCNV (Wang et al., 2007) and QuantiSNP (Colella et al., 2007), were developed to infer CNVs directly from SNP array data. Winchester et al. (2009) provided a comprehensive summary of existing algorithms for CNV inferred from SNP array. CNV inferred by SNP array are not as accurate as aCGH and usually cannot be used to detect small variants (<30 Kb) (Zhang et al., 2014) as the density of SNP probes is sparse for most genomic regions. However, the advantage is that CNV analysis can be performed directly on existing array data that were originally intended for GWAS. In addition to CNVs, SNP-array analysis is also possible to infer certain balanced SV, such as inversions from linkage disequilibrium pattern (Cáceres et al., 2012).

### Short-read sequencing

**Figure 2:**
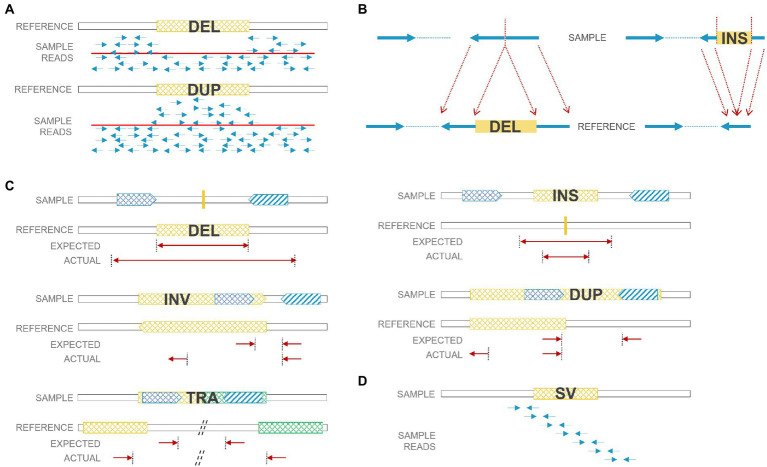
SV detection strategies using short reads. **(A)** Read-depth-based method: deleted regions have low coverage; duplicated regions have high coverage. **(B)** Split-read-based method: deleted/inserted regions can be detected by split reads. **(C)** Paired-end-read based method: paired reads from deletions/duplications/insertions/inversions/translocations have unexpected insert size or orientation. **(D)** De nova sequence assembly. DEL: deletion; DUP: duplication; INS: insertion; INV: inversion; TRA: translocation.

As next-generation sequencing (NGS), especially paired-end sequencing, has become widely affordable and available, researchers have been able to detect SVs in a finer resolution and explore balanced SVs, including insertions and inversions that were often ignored before. However, the accuracy of calling SVs and the precision of breakpoints are still inadequate due to certain limitations of the short-read technology. There were numerous algorithms developed for SV detection, and a review of SV calling algorithms for NGS was summarized previously (Lin et al., 2015; Guan and Sung, 2016). Technically, there are four main strategies for SV calling: read depth, paired-end reads, split reads, and *de novo* assembly (Figure 2). 1. Read-depth-based algorithms mainly focus on CNV identification through comparison of the read depth of a specific region and the read depth of its surroundings (or average depth); 2. Paired-end-read-based algorithms can identify CNVs as well as insertions, inversions, and translocations. It detects abnormal insert size, alignment orientation (for duplications and inversions), or alignment locations (for translocations) between the two ends of a paired-end read; 3. Split-read-based methods detect SVs from reads that cross SV breakpoints. Along with breakpoints, reads can be split and mapped against the reference genome encompassing SVs. Therefore, theoretically, split-read methods can provide single-nucleotide resolution for SV breakpoints. However, breakpoints regularly harbor short repeats that bring challenges when performing split-read strategy for SV detection. Also, higher coverage is needed to accumulate enough split reads for SV calling; 4. *De novo* sequence assembly reassembles contigs and then compares contigs to references. It is the most accurate and not biased by reference sequences, but it needs high coverage (or prone to assembly errors) and induces high computational cost. To increase the sensitivity in SV calling, several strategies were usually combined together to form a hybrid algorithm, such as LUMPY (Layer et al., 2014) and Manta (Chen et al., 2016).

### Long-read sequencing and other technologies

Other than NGS, a few new methods and techniques can be applied to a more accurate SV detection, including single-molecule long-read sequencing by Pacific Biosciences and Oxford Nanopore Technologies, as well as new platforms such as Illumina Infinity. Particularly, long-read sequencing can help to solve regions that have few or low-mappable reads due to high GC content or other chemical issues, and also regions that have a low mapping quality due to repetitive sequences (Jain et al., 2018; Ebbert et al., 2019). Despite higher error rate on each nucleotide, long-read sequencing can provide higher mappability in those “problematic regions,” have ability to span entire SVs, and offer sufficient long split reads to lead a better tool for SV detection. Overall, long reads and phased genome generated by long-read sequencing enable more precise detection of breakpoints and more SVs compared to short-read sequencing (De Coster and Van Broeckhoven, 2019).

Futhermore, optical mapping detects SVs from *de novo* assemblies created by imaging intact single molecules of native-state DNA (Chan et al., 2018). Synthetic long-read techniques (including Illumina TruSeq) generate low-error local assembly of short-read data through specific library preparations (McCoy et al., 2014; Zheng et al., 2016) and can better resolve repetitive regions and phase haplotypes for SV detection. By tagging nascent strand using bromodeoxyuridine (thymidine analog) during replication, Strand-seq is able to sequence individual DNA strand through removing the nascent strand following photolysis induced by UV light (Falconer et al., 2012; Ho et al., 2020). The directionality obtained from single strand sequencing makes Strad-seq particularly well-suited for inversion discovery and haplotype phasing. Additionally, Hi-C can be used to infer interchromosomal translocations and large intrachromosomal SVs by abnormal chromatin interactions around the SV breakpoints (Lieberman-Aiden et al., 2009; Wang et al., 2022).

## Copy number variants in Alzheimer’s disease

### Overall burden of CNVs in AD

In autism and schizophrenia, many studies have confirmed large (>100 Kb) rare (<1%) CNV (usually deletion) burden in patients versus controls (Walsh et al., 2008; Kirov et al., 2009a; Buizer-Voskamp et al., 2011; Kushima et al., 2018; Maury et al., 2022). However, there were mixed results in the evaluation of overall CNV burden in AD studies. Some earlier analysis based on SNP array found no significant difference in CNV rate and CNV size between AD cases and controls (Ghani et al., 2012; Swaminathan et al., 2012b). Guffanti et al. (2013) found overrepresentation of large heterozygous deletions in cases (*p*-value <0.0001) using 459 AD/mild cognitive impairment (MCI) cases and 181 health controls in ADNI. From CNVs called on NGS data, even though no significant difference in CNV count or length between AD cases and controls were found (Lee et al., 2021; Ming et al., 2021), non-Hispanic-white AD cases showed more duplications, and Hispanic AD cases showed larger deletions when burden analysis was stratified by ethnicity (Lee et al., 2021). Due to the fact that only large CNVs can be detected from SNP array and false positive CNVs detection is a major concern for short read data, future studies involving more robust and comprehensive CNV callings are needed to clarify the overall burden of CNVs in AD.

### CNVs in early-onset AD

For early-onset familial AD, hundreds of pathogenic mutations in *APP*, *PSEN1*, and *PSEN2* have been discovered. In a study from France that included 12 unrelated individuals with autosomal dominate early-onset AD (EOAD), 70 unrelated individuals with familial LOAD, and 100 health controls, the *APP* duplications ranging from 0.58 to 6.37 Mb were found in five individuals with autosomal-dominant EOAD (Table 1; Rovelet-Lecrux et al., 2006). In other studies of Europeans, however, the *APP* duplication was identified as a relatively rare cause of autosomal dominant EOAD (Sleegers et al., 2006; Blom et al., 2008; Hooli et al., 2012). In samples with Japanese ancestry, two unrelated early-onset familial AD families were found to harbor the *APP* duplication (with SV size about 4.2 and 0.7 Mb) among 25 families with familial AD and 11 sporadic EOAD cases (Kasuga et al., 2009). Overall, duplications in *APP* are rare, but indeed an important cause of autosomal dominant EOAD.

**Table 1:** Copy number variants associated with Alzheimer’s disease.

Gene	Location	Size	Consequence	Method	CNV	Sample	Evidence	References
APP	21q21.3	0.57–6.37 Mb	Gene duplication	PCR	Duplication	ADEOAD: 12, familial LOAD: 70, control: 100	CNV in 5 ADEOAD families	Rovelet-Lecrux et al. (2006)
PSEN1	14q24.2	-	Deletion of exon 9	PCR	Deletion	1 family: 60 individuals	CNV in 17 of 60	Crook et al. (1998)
CHRNA7	15q13.3	500 Kb	Gene duplication	SNP Array	Duplication	AD: 331, control: 368	*p* = 0.053 (six cases, one control)	Heinzen et al. (2010)
CHRFAM7A	15q13.2	30 Kb	Deletion of multiple exons	SNP Array	Deletion	AD: 222, MCI: 136, control: 143	Case only	Swaminathan et al. (2011)
CYFIP1	15q11.2	470 Kb	Gene duplication	SNP Array	Duplication	AD: 559, control: 554	*p* = 0.037 (10 cases, three controls)	Ghani et al. (2012)
CR1	1q32.2	18 Kb	Additional C3b/C4b binding sites	PCR	Duplication	AD: 1039, control: 844	*p* < 0.05	Brouwers et al. (2012)
SULT1A3/4	16p11.2	–	Gene duplication	PCR	Duplication	AD: 91, control:172	*p* = 0.011	Butcher et al. (2018)
AMY1A	1p21.1	–	Gene duplication	PCR	Duplication	AD: 247, control: 5175	*p* < 0.05	Byman et al. (2020)
CPNE4	3q22.1	3.6 Kb	Intragenic deletion	SNP Array	Deletion	AD: 375, control: 192	FDR = 0.033	Szigeti et al. (2013)
APC2	19p13.3	–	–	SNP Array	Duplication	AD (psychosis): 496, AD (intermediate psychosis): 639, AD: 156	7.20 × 10^−10^	Zheng et al. (2015)
CREB1	2q33.3	8 Kb	Upstream of CREB1 (Pax-6 binding site)	Expression/SNP Array	Deletion	AD: 22, control: 15 (DEG); AD: 1230, control: 936 (CNV)	*p* (DEG) < 0.001; *p* (CNV) = 0.008	Li et al. (2012)
A2BP1, ABAT, CDH2, CRMP1, DMRT1, EPHA5, EPHA6, ERMP1, EVC, EVC2, FLJ35024, VLDLR				SNP Array	CNVs	261 families: 1,009 individuals	Case only	Hooli et al. (2014)
CSMD1, HNRNPCL1, SLC35F2				SNP Array	CNVs	AD: 222, MCI: 136, control: 143	*p* < 0.05	Swaminathan et al. (2011)
ATXN1, HLA-DPB1, RELN, DOPEY2, GSTT1				SNP Array	CNVs	AD: 711, control: 171	Case only	Swaminathan et al. (2012b)
CHRFAM7A, RELN, DOPEY2, HLA-DRA				SNP Array	CNVs	AD: 728, control: 438	*p* < 0.05	Swaminathan et al. (2012a)
MAGI1, NLRP8, KLK6, MEOX2, SLC30A3, FPR2, SVOP				aCGH	CNVs	ADEOAD: 23, sporadic EOAD: 12, other AD: 912, control:1078	EOAD only singletons	Rovelet-Lecrux et al. (2012)

AD, Alzheimer’s Disease; ADEOAD, Autosomal Dominant Early-Onset Alzheimer’s Disease; CNVs: Copy Number Variation; DEG, Differentially Expressed Gene; EOAD, Early-Onset Alzheimer’s Disease; LOAD, Late-Onset Alzheimer’s Disease; MCI, Mild Cognitive Impairment.

Other than the widely recognized *APP* duplications, deletions of exon 9 in *PSEN1* have been discovered in families affected by a variant of AD with spastic paraparesis and unusual plaques (Table 1; Crook et al., 1998; Smith et al., 2001), and 10 novel CNVs overlapping a set of genes, including *A2BP1*, *ABAT*, *CDH2*, *CRMP1*, *DMRT1*, *EPHA5*, *EPHA6*, *ERMP1*, *EVC*, *EVC2*, *FLJ35024*, and *VLDLR*, were found to co-segregate with disease status from 261 early-onset familial AD families (Hooli et al., 2014).

### Array-based analysis

Using CNVs inferred from SNP array, Heinzen et al. (2010) reported a large 500 Kb duplication located at 15q13.3 in 6 of 276 cases and 1 of 322 controls (Table 1, *p* = 0.053). The duplicated region covers entire *CHRNA7* gene and was confirmed to be associated with schizophrenia and epilepsy (Stefansson et al., 2008). Interestingly, Swaminathan et al. (2011) also identified a 30 Kb deletion from ADNI that overlaps with *CHRFAM7A*, which is *CHRNA7* (Exons 5–10) and *FAM7A* (Exons A–E) fusion, and appears in 4 of 471 AD/MCI cases but not in 184 health controls (Table 1). In the same study, CNVs in *CSMD1*, *HNRNPCL1*, and *SLC35F2* were case-specific with a nominal *p* < 0.05, though no significant signal was reported after adjusting for multiple tests (Table 1; Swaminathan et al., 2011). In 2012, Swaminathan et al. (2012b) extended the study and performed similar analysis on 882 unrelated non-Hispanic Caucasian participants in the National Institute of Aging LOAD/National Cell Repository for AD (NIALOAD/NCRAD) Family Study. Again, no genome-wide significant signal was observed. However, CNVs in five AD candidate genes (*ATXN1*, *HLA-DPB1*, *RELN*, *DOPEY2*, and *GSTT1*) only showed in cases in both NIALOAD/NCRAD and ADNI (Table 1). When TGen cohort consisting of 728 cases and 438 controls was used to validate the results from NIALOAD/NCRAD and ADNI, CNVs in AD candidate genes, including a number of previously reported regions (*CHRFAM7A*, *RELN*, and *DOPEY2*) as well as a new gene (*HLA-DRA*), were identified (Table 1; Swaminathan et al., 2012a). Using the four algorithms to detect CNVs from 1,103 samples, Ghani et al. (2012) identified a 470 Kb duplication in 15q11.2 (encompassing *TUBGCP5*, *CYFIP1*, *NIPA2*, *NIPA1*, and *WHAMML1*) appearing in 10 cases (2.6%) and three controls (0.8%) with nominal significance (*p* = 0.037), and indicated that CNVs in *NRXN1*, *CNTNAP2*, *PTPRD*, *NDUFAF2*, and *CNTN6* were also worthy of further study (Table 1). Notably, for the deletion in *NRXN1*, several other reports suggested that it could increase the risk of developing Schizophrenia (Kirov et al., 2009b; Maury et al., 2022). Combining gene expression with dosage information from SNP array, an 8 Kb deletion containing a PAX6-binding site on the upstream of *CREB1* was associated with AD (Table 1; Li et al., 2012). In a study comparing 33 EOAD cases with 212 LOAD cases and 1,078 controls using high-resolution aCGH, seven singleton CNVs were reported from EOAD samples (Table 1; Rovelet-Lecrux et al., 2012).

None of genes mentioned above reached genome-wide significance after adjusting for multiple tests. Two studies found genome-wide significant CNVs using alternative phenotypes (Age of onset and AD with psychosis): Szigeti et al. (2013) identified five significant regions (FDR < 0.05) ranging from 3.6 to 24.8 Kb that were related to the age of onset in AD, including a intragenic deletion in *CPNE4* (Table 1). Using 496 AD cases with psychosis, 639 AD cases with intermediate psychosis and 156 AD cases without psychosis, Zheng et al. (2015) found a duplication (odds ratio = 0.42; *p* = 7.2 × 10^−10^) in the *APC2* that is protective against developing psychosis in AD (Table 1).

### Whole-genome-sequencing-based analysis

Just like studies done on SNP arrays, association analysis of SVs detected from NGS yielded no genome-wide significant signal. There could be a few explanations for that. First, the sample size of studies using NGS is usually smaller than SNP arrays, although more SVs are expected from using NGS. Second, even joint genotyping can increase sample sizes, joint genotyping is tough to be properly done for SVs due to lack of well-aligned breakpoints. Third, repetitive genomic regions cause artificial alignments that would lead to false positive SV detection and introduce noises of association analysis.

While association between SVs and AD status reveals no significant signal, using 1,411 samples in MSBB and ROSMAP, Ming et al. (2021) found that the AD-specific CNVs showed distinct functional annotations compared to MCI-specific and normal-specific CNVs, such as glucuronosyltransferase activity, cellular glucuronidation, and neuron projection. Moreover, Vialle et al. (2022) performed an SV-xQTL analysis and identified more than 3,200 SVs that were correlated with histone modifications, gene expression, splicing, or protein abundance in postmortem brain tissues, providing a valuable resource for functional study of SVs.

Single-cell whole genome sequencing (scWGS) has been applied to study the aneuploidy in AD patients (van den Bos et al., 2016). Since neurons are post-mitotic, earlier studies of increased overall aneuploidy in AD patients were based on the analysis of metaphase cells in whole peripheral blood (Ward et al., 1979). The application interphase FISH allowed detection of aneuploidy in neuronal cells in human brains. However, interphase FISH can only target a limited number of chromosomes in one cell and is intrinsic noisy, and thus studies often showed different results. Iourov et al. (2009) reported chromosome 21-specific aneuploidies in the cerebral cortex of AD patients, while other studies indicated aneuploidies caused by increased tetraploid neurons in AD patients due to a full S phase without initiation of mitosis (Yang et al., 2001; Mosch et al., 2007). With the advent of scWGS, all chromosomes in a cell can be analyzed, and each chromosome in a cell was probed thousands of times by different sequencing reads. Moreover, unlike interphase FISH, analysis of aneuploidy by scWGS would not be affected by tissue sectioning or other possible causes of artifacts. van den Bos et al. (2016) used scWGS for studying aneuploidies of neuronal cells and found no significant difference between AD cases and controls. Besides, giving the wide application of scWGS in human genetics and disease etiology (Zhang et al., 2019; Porubsky et al., 2021; Salehi et al., 2021), the discovery of somatic SVs in single-cell resolution in brains of AD patients would definitely yield valuable new insights about AD pathogenesis.

### Targeted analysis of CNVs on specific genes in AD

Complement receptor gene 1 (*CR1*) was identified and replicated as a causal gene for AD by several GWASs (Harold et al., 2009; Lambert et al., 2009). However, the specific mechanism of *CR1* disruption in AD remains unclear. The significant SNP (rs4844610) was in an LD block which spans nearly the entire gene *CR1*, except for the first and last exons. Within the LD block, there is an 18 Kb low-copy repeat (LCR) which represents the same signal as the SNP by conditional regression analysis (Table 1; Brouwers et al., 2012). Generally, LCRs usually lead to genome instability due to non-allelic homologous recombination (Erdogan et al., 2006). In CR1 protein, the LCR can affect protein function by creating additional binding sites for complement components C3b and C4b (Brouwers et al., 2012). Therefore, the LCR CNV is likely to explain the significant association within this LD block.

SULT1A3/4 are important enzymes in the metabolism of catecholamines which are involved in many neurodegenerative diseases. Butcher et al. (2018) evaluated association of copy number of *SULT1A3/4* and the risk of AD and Parkinson’s Disease (PD; Table 1). For those individuals with less than four copies of *SULT1A3/4*, their ages of onset for AD were earlier, and they were more likely to develop AD (odds ratio = 1.69), but the association with PD was not significant.

Byman et al. (2018) found evidences supporting the role of α-amylase involvement in AD pathology. Subsequently, they further studied relationship between *AMY1A* copy number with AD status and memory performance (Byman et al., 2020). There is no significant difference in *AMY1A* copy number between cases and controls. However, individuals with high copy number of *AMY1A* (≥10) showed significantly lower hazard ratio compared to reference (Table 1). The full list of CNVs associated with AD is listed in Table 1.

## Other structural variants in neurodegenerative diseases

### Inversions, insertions, and complex SVs

Compared to CNVs, insertions, inversions, and complex SVs are less studied due to the limitation of array-based analysis, although they are important causal factors in the pathogenesis of neurodegenerative diseases. Sato et al. (2009) identified a 2.5–3.8 Kb insertion containing a long (TGGAA)n stretch in patients with spinocerebellar ataxia type 31 (Table 2); the length of the insertion was inversely correlated with age onset of the disease. Prion diseases are often caused by a conversion of normal prion protein (PrP^C^) into a proteinase K resistant form of PrP (PrP^Sc^). Insertion of 2–12 octapeptide repeats between codon 51–91 of *PRNP* can cause genetic form of prion disease (Table 2; Rossi et al., 2000; Moore et al., 2001; Kim et al., 2018). These octapeptide insertion can cause rapid binding between PrP molecules, therefore increasing the rate of PrP^Sc^ formation (Moore et al., 2006). In a study of SVs in amyotrophic lateral sclerosis (ALS) genes, an inversion in the *VCP* gene and an insertion in *ERBB4* gene were identified to be able to increased risk of ALS besides the repeat expansion in *C9ORF72* from whole genome sequencing of 6,500 individuals (Table 2; Al Khleifat et al., 2022).

**Table 2:** Other structural variants in neurodegenerative diseases.

Type	SVs	Location	Gene	Risk allele	Inheritance	Disease
STR	(CAG)n	4p16.3	HTT	>39	Autosomal dominant	Huntington’s disease
STR	(CTG)n	16q24.2	JPH3	>39	Autosomal dominant	Huntington’s disease-like 2
STR	(CGG)n	Xq27.3	FMR1	>200	X-linked dominant	Fragile X syndrome
STR	(GAG)n	12p13.31	ATN1	>48	Autosomal dominant	Dentatorubral–pallidoluysian atrophy
STR	(CAG)n	6p22.3	ATXN1	>39	Autosomal dominant	Spinocerebellar ataxia type 1
STR	(CAG)n	12q24.12	ATXN2	>36	Autosomal dominant	Spinocerebellar ataxia type 2
STR	(CAG)n	14q32.12	ATXN3	>60	Autosomal dominant	Spinocerebellar ataxia type 3
STR	(CAG)n	19p13.2	CACNA1A	>18	Autosomal dominant	Spinocerebellar ataxia 6
STR	(CAG)n	3p14.1	ATXN7	>36	Autosomal dominant	Spinocerebellar ataxia 7
STR	(CTG)n	13q21.33	ATXN8OS	>53	Autosomal dominant	Spinocerebellar ataxia 8
STR	(ATTCT)n	22q13.31	ATXN10	>800	Autosomal dominant	Spinocerebellar ataxia 10
STR	(CAG)n	5q32	PPP2R2B	>54	Autosomal dominant	Spinocerebellar ataxia 12
STR	(CAG/CAA)n	6q27	TBP	>40	Autosomal dominant	Spinocerebellar ataxia 17
STR	(GGCCTG)n	20p13	NOP56	>650	Autosomal dominant	Spinocerebellar ataxia 36
STR	(GAA)n	9q21.11	FXN	>70	Autosomal recessive	Friedreich’s ataxia
STR	(GGGGCC)n	9p21.2	C9ORF72	>30	Mainly in familial ALS	Amyotrophic lateral sclerosis
STR	(CAG)n	12q24.12	ATXN2	>26	Increasing risk	Amyotrophic lateral sclerosis
STR	(CA)n	8q21.13	STMN2	24	Increasing risk	Amyotrophic lateral sclerosis
STR	(GGGGCC)n	9p21.2	C9ORF72	>30	Mainly in familial FTD	Frontotemporal dementia
STR	(CAG)n	12q24.12	ATXN2	>36	Autosomal dominant	Parkinson’s disease
STR	(GGGGCC)n	9p21.2	C9ORF72	>60	Rare cause	Parkinson’s disease
STR	(AAGGG)n	4p14	RFC1	>144	Rare cause	Parkinson’s disease
STR	repeat expansion in ATXN3, ATXN10, and TBP				In PD patients	Parkinson’s disease
STR	(CAG/CAA)n	6q27	TBP	>38	Increasing risk	Multiple system atrophy
VNTR	25 bp motif	19p13.3	ABCA7	>228	Increasing risk	Alzheimer’s disease
VNTR	14 bp motif	11p15.5	INS	Class III allele homozygotes	Earlier onset	Alzheimer disease
VNTR	69 bp motif	18q21.31	WDR7	–	Increasing risk	Amyotrophic lateral sclerosis
VNTR	21 bp motif	2q35	XRCC5	2R allele	Increasing risk	Multiple Sclerosis
VNTR	86 bp motif	2q13	IL1RN	IL1RN*2 allele	Earlier onset	Wilson’s disease (neuropsychiatric form)
SVA	SVA	2q14.3	BIN1	–	Increasing risk	Alzheimer’s disease
SVA	SVA	16p11.2	BCKDK	–	Increasing risk	Parkinson’s disease
SVA	(CCCTCT)n	Xq13.1	TAF1	>34	X-linked	X-linked dystonia-parkinsonism
SVA	SVA	17q21.31	MAPT	–	Increasing risk	Progressive supranuclear palsy
Alu	PolyT	19q13.32	TOMM40	18–30	Increasing risk	Alzheimer disease
Insertion	Octapeptide repeat insertion	20p13	PRNP	2–12 octapeptide repeats	Autosomal dominant (>4 repeats)	Prion disease
Insertion	(TGGAA)n	16q21	BEAN1	Repeat insertion	Autosomal dominant	Spinocerebellar ataxia 31
Insertion	Insertion	2q34	ERBB4	–	Increasing risk	Amyotrophic lateral sclerosis
Inversion	Inversion	9p13.3	VCP	–	Increasing risk	Amyotrophic lateral sclerosis
Complex SVs	~1 Mb haplotype	17q21.31	MAPT	H1	Increasing risk	Progressive supranuclear palsy

Non-canonical or complex SVs are usually hard to decipher as they are typically composed of multiple breakpoint junctions and cannot be characterized as a single canonical SV type, but they may harbor important disease-causing variants. For example, the region of 17q21.31 containing *MAPT* has two major haplotypes: H1 and H2. The H2 haplotype is characterized by a ~900 Kb inversion flanked by two duplication blocks, and tagged by a 238 bp deletion between exons 9 and 10 of *MAPT* and a few SNPs inside (Baker et al., 1999). The H2 haplotype is positively selected in Europeans and is completely absent or extremely rare in other ethnic groups (Pittman et al., 2006). Nearly all individuals carrying the H2 haplotype were in the same structural form (H2.α2.γ2), indicating it may derive from a single founder (Boettger et al., 2012). In contrast, the H1 haplotype is more variable and exists in all ethnic groups. As for the origin of H1 and H2, one study reported that they do not follow a precursor-product relationship and cannot be derived directly from each other (Hardy et al., 2005), while another study preferred a H2-like ancestor (Zody et al., 2008).

Many studies have reported the association of the inverted H2 haplotype with reduced risk of a range of neurodegenerative disease, including progressive supranuclear palsy (PSP) (Table 2; Baker et al., 1999), frontotemporal disorders (FTD; Baker et al., 1999), AD (Allen et al., 2014), and PD (Zabetian et al., 2007). Due to the important role of *MAPT* in tau pathology, variants in *MAPT* were among the most studied ones in this complex region. *MAPT* can produce six major tau isoforms by the alternative splicing of exons 2, 3, and 10. Alternative splicing of exon 10 can lead to imbalance of three microtubule-binding repeats (3R) tau (exclusion of exon 10) and four microtubule-binding repeats (4R) tau (inclusion of exon 10) ratio which were associated with several tauopathies in brain (Pittman et al., 2006). It was showed that H1 expresses more *MAPT* exon 10 mRNA compared to H2 (Caffrey et al., 2006), conforming to the increased 4R tau isoforms in PSP (Arai et al., 2001). The 238 bp deletion between exons 9 and 10 was considered to influence the alternative splicing of exon 10, therefore, affecting the risk of developing PSP (Baker et al., 1999). In addition, four highly correlated SVs [three deletions and one SINE-VNTR-Alu (SVA)] tagging H1/H2 haplotype were identified and may regulate nearby gene expression (Vialle et al., 2022).

Other than variants in LD with the H2/H1 haplotype, several deleterious rare SVs were also identified in this region. Microdeletions (~600 Kb) encompassing *MAPT* and *CRHR1* in 17q21.37 were identified in three individuals with intellectual disability (Koolen et al., 2006). A partial deletion of exons 6 to 9 of *MAPT* causing a truncated tau isoform was detected in a FTD patient (Rovelet-Lecrux et al., 2009). Duplications (~460 Kb) spanning *MAPT* were found in two PSP patients from a total of 283 PSP patients (Chen et al., 2019). A list of neurodegenerative diseases caused by insertions, inversions, and complex SVs were displayed in Table 2.

### Short tandem repeats and variable number of tandem repeats

Short-tandem repeats (STRs) are repeating DNA sequences of 2–6 base pairs in length, which are often referred as microsatellites or simple sequence repeats (SSRs) when used in different circumstances. Many neuropathological diseases were caused by STRs, such as Huntington’s disease, Fragile X syndrome, Dentatorubral-pallidoluysian atrophy, and several spinocerebellar ataxias (Ryan, 2019). Huntington’s disease is a progressive disorder that interrupts mood, movement, and intellectual abilities. It is an autosomal dominant disease caused by a trinucleotide CAG repeat expansion (from 36 to 120 CAG repeats in patients) in the *HTT* (Huntingtin) gene, which leads to a polyglutamine stretch causing dysfunction in the HTT protein (Table 2; Penney et al., 1997). In addition to CAG repeats in *HTT*, increased copy number of *SLC2A3* can increase the level of GLUT3, therefore, delaying the age onset of Huntington’s disease. The experimental validation showed that increased dosage of *Drosophila melanogaster* homologue Glut1 ameliorated Huntington’s disease related phenotypes in fruit flies (Vittori et al., 2014). Another CTG repeat expansion in *JPH3* was causal for a similar autosomal dominantly inherited disease, i.e., Huntington disease-like 2 (Table 2; Stevanin et al., 2003).

Fragile X syndrome is a rare neurodegenerative disease that causes a range of developmental problems, including learning disabilities, and cognitive impairment. Normally, people have less than 45 CGG repeats in the *FMR1* gene, while patients with Fragile X syndromes have more than 200 CGG repeats, causing failure in making FMPR protein (Table 2; Kremer et al., 1991). Dentatorubral-pallidoluysian atrophy is a neurodegenerative disease characterized by variable combinations of myoclonus, epilepsy, ataxia, choreoathetosis, and dementia, and its clinical presentation correlates with the number of CAG repeats in *ATN1* (Table 2; Koide et al., 1994).

Ataxias were characterized by gait ataxia and other cerebellar signs due to progressive loss of nerve cells. Hereditary ataxias can be grouped into three categories: the autosomal dominant ataxias, also called spinocerebellar ataxias (SCA), autosomal recessive cerebellar ataxias (ARCA), and X-linked ataxias. SCA is the most common form of hereditary ataxias, and there are more than 30 genetic causes for SCA (Shakkottai and Fogel, 2013), many of which were caused by STRs in genes. For example, SCA1/SCA2/SCA3 are caused by an expanded CAG trinucleotide repeat in the *ATXN1*/*AXTN2*/*AXTN3* gene (Table 2; Banfi et al., 1994; Bürk et al., 1996; Damji et al., 1996; Stevanin et al., 1996). Simple repeat CAG/CAA (>38) in *TBP* is the cause of SCA17 (Nakamura et al., 2001) and is associated with increased risk of multiple system atrophy (Wernick et al., 2021). Friedreich’s ataxia is one of the most common forms of ARCA. Patients with Friedreich’s ataxia were caused by abnormal copy number of GAA trinucleotide repeat in *FXN* gene. Normally, there are less than 30 GAA repeats in *FXN*, while GAA repeats 70 to more than 1,000 times in patients with Friedreich’s ataxia (Table 2; Campuzano et al., 1996; Anheim et al., 2012). The higher copy number of GAA was associated with more severe and faster evolving disease form (Bhidayasiri et al., 2005). Other than STRs, CNVs in *SACS*, *SYNE1*, *ADCK3*, and *SETX* were found to be potential causal genes for ARCA (Campuzano et al., 1996; Anheim et al., 2012; Cheng et al., 2021).

Currently, the association of expanded GGGGCC repeat in *C9ORF72* with FTD and ALS (DeJesus-Hernandez et al., 2011) has been widely recognized (Table 2). Besides mutations in *SOD1*, *TARDBP*, and *FUS,* GGGGCC repeat in *C9ORF72* is the main cause of ALS and accounts for 30–40% of familial ALS and 7% of sporadic form of ALS (Al-Chalabi et al., 2012). In FTD, pathogenic repeat expansion in *C9ORF72* accounts for 20–30% of familial form and about 6% of sporadic form of FTD (DeJesus-Hernandez et al., 2011), and is the most common cause of disease besides pathogenic mutations in *GRN* and *MAPT* (Sirkis et al., 2019). The *C9ORF72* repeat also showed segregation with AD status in three families (Harms et al., 2013). In addition to the *C9ORF72* repeat, the number of CA repeat in *STMN2* (Theunissen et al., 2021) and CAG repeat in *ATXN2* (Elden et al., 2010) were associated with risk and age-of-onset of ALS.

In PD, mutations in 18 *PARK* genes have been found as the main cause of familial PD (Klein and Westenberger, 2012). For example, exonic deletions in *Parkin* gene were the most common mutations in families with autosomal recessive parkinsonism (Taghavi et al., 2018), and *SCNA* duplication can lead to autosomal dominant PD (Singleton et al., 2003; Konno et al., 2016). Besides, STRs in a few ataxia genes can be a rare cause of PD. CAG repeat expansion in SCA2 gene *ATXN2* is also the cause of autosomal dominant PD (Table 2; Charles et al., 2007). The biallelic AAGGG repeat expansion in *RFC1* is a common cause of late-onset ataxia (Cortese et al., 2019) and is likely to be a rare cause of PD (Kytövuori et al., 2022). Repeat expansions in SCA3, SCA10, and SCA17 have been described in patients with PD as well (Park et al., 2015; Schüle et al., 2017). In addition, the GGGGCC repeat expansion in *C9ORF72* is also a rare cause of PD (Table 2; Lesage et al., 2013).

As a type of minisatellite with sequence repeats vary between individuals, variable number of tandem repeats (VNTRs) are usually considered as longer cousins of STRs. A 69 bp VNTR in *WDR7* was found to be enriched in sporadic ALS patients (Table 2; Course et al., 2020). VNTR in *IL1RN* intron 2 was related to earlier Wilson’s disease onset, particularly among patients with neuropsychiatric form of the disease (Table 2; Gromadzka and Członkowska, 2011). Frequency of 2R allele of *XRCC5* gene was significant different between multiple sclerosis (MS) patients and controls (Table 2; Jahantigh et al., 2017). Individuals with homozygous *INS* class III alleles (characterized by 141–209 repeats of 14 bp motif) showed earlier onset of AD (Table 2; Majores et al., 2002). An 25 bp intronic VNTR expansion in *ABCA7* was associated with GWAS SNP (rs3764650) and enriched in AD patients (Table 2, odds ratio = 4.5 (1.3–24.2); De Roeck et al., 2018).

The traditional method to detect STRs/VNTRs is low throughput as it involves performing polymerase chain reaction on target regions and gel electrophoresis (DeJesus-Hernandez et al., 2011). With the availability of sequencing data, detecting STRs/VNTRs in a large scale is practical. Currently, various algorithms have been developed for STR/VNTR detection from sequencing data, e.g., popSTR (Kristmundsdóttir et al., 2017), GangSTR (Mousavi et al., 2019), STRetch (Dashnow et al., 2018), and ExpansionHunter (Dolzhenko et al., 2019) for STR detection, and VNTRseek (Gelfand et al., 2014), adVNTR(Bakhtiari et al., 2018) and code-adVNTR (Park et al., 2022) for VNTR detection. A few reviews (Gymrek, 2017; Halman and Oshlack, 2020) have provided a comprehensive list of tools available for STR detection from sequencing data. Considering the successful application of profiling STRs/VNTRs associated with disease status and gene expression (Bakhtiari et al., 2018; Fotsing et al., 2019; Mousavi et al., 2019; van der Sanden et al., 2021), studying STRs/VNTRs in a large cohort of AD patients may yield new discovery to AD genetics. A list of neurodegenerative diseases caused by STRs/VNTRs are displayed in Table 2.

### Transposable elements related SVs

Transposable elements (TEs) are DNA sequences that can move between different genomic positions. About 44% of the human genome is consisted of transposons: 20% long interspersed element (LINEs), 13% short interspersed elements (SINEs), 8% long terminal repeats (LTR) retro-transposons, and 3% DNA transposons (Mills et al., 2007). More importantly, TEs can serve as an active mutagen in the human genome, causing insertions, deletions, duplications, inversions, and translocations (Beck et al., 2011). It has been reported that TEs are activated in AD, causing genomic instability (Guo et al., 2018). TEs are also widely involved in a range of other neurodegenerative diseases (Tam et al., 2019), implicating potential pathogenic transposon-induced SVs underlying the genetic basis of neurodegenerative disorders.

One class of TEs, called SINE-VNTR-Alu (SVA), is of particular interest since it naturally harbors VNTRs with gene-regulatory functions inside its body. It was reported that 82% of structurally variable SVAs were around 50 Kb of a transcription start site, indicating their role as a transcriptional regulatory element in disease pathogenesis (van Bree et al., 2022). In the same study, a few SVAs in the LD blocks of GWAS SNP signals for AD, PD, MS, and ALS were identified; the deletion of SVAs around *BIN1* (AD gene) and *BCKDK* (PD gene) alters epigenome and nearby gene expression (Table 2; van Bree et al., 2022). X-linked dystonia-parkinsonism (XDP) patients carry a 294 Kb identical founder haplotype harboring a few sequence variants. Particularly, the number of CCCTCT repeat inside the SVA element in this region displayed highly significant inverse correlation with age of XDP onset, suggesting this repeat could be the causative variant (Table 2; Bragg et al., 2017). In a study of SVAs in PD, no significant association with disease risk was reported; however, one SVA inside the H1 haplotype on chromosome 17 was reported and found to be associated with the expression of multiple genes at this locus (Pfaff et al., 2021).

In AD, there is evidence that primate-specific Alu retrotransposons repeatedly inserted into the intron of *TOMM40* (Table 2; Larsen et al., 2017), which has been implicated in the pathogenesis of AD by numerous GWAS studies. The deoxythymidine homopolymer repeat (rs10524523), a part of Alu mobile element monomer, is inserted into the intron 6 of *TOMM40*, and is usually in the same haplotypes as *APOE* alleles with long repeats (18 bp <and <30 bp) corresponding to *APOE* E4 and short repeats (≤18 bp) or very long repeats (≥30 bp) corresponding to *APOE* E2/E3 in Caucasians (Crenshaw et al., 2013). A list of neurodegenerative diseases caused by TE related SVs were displayed in Table 2.

## Discussion

From GWASs in the past decade, dozens of SNPs with small effects were associated with AD. Among of those identified SNPs, *APOE*4/2 remained as the most powerful alleles in predicting the risk of LOAD. *APOE* alleles alone can reach an AUC (Area Under the Curve) of 0.70 in predicting AD, while the best AUC is 0.61 for all other SNPs combined (Leonenko et al., 2021). SVs are major genetic mutations residing in the genome besides SNPs. Extending current genetical analysis further into the field of SVs would definitely help improve the polygenic prediction of AD risk, therefore, facilitating earlier diagnosis and treatment before irreversible pathological damages were done. Moreover, it was pointed out that the most important limiting factor in the translation of knowledge from genetics to drugs is the lack of good models for AD (Sierksma et al., 2020). Potential mechanism of actions in disease etiology identified by SVs might lead to new animal models and drug targets for the treatment of AD.

So far, the *APP* duplication is the only structural variation with sufficient supports for directly causing AD. Other than that, a few candidate SVs (Table 1) associated with increased risk of AD are identified. The reasons for the lack of new discoveries are multifold. First, only large CNVs can be detected on SNP array. Those large CNVs tend to be rare and are not likely to reach statistical significance without sufficient sample size. Second, even though smaller SVs can be obtained from short-read sequencing data, reliable read mapping for SV calling from short reads still remains challenging since half of the human genome is made of repetitive sequences (Lander et al., 2001). Third, unlike SNPs which affect single locus, SVs are usually represented by a range that may have different breakpoints in different individuals, therefore, increasing the difficulties in analyzing SVs.

In the future, with the revolution and refinement in sequencing techniques, particularly, long sequencing techniques, we believe that we would be able to characterize SVs in a large sample size accurately, exhaustively, and cost-efficiently. The issue of low mappability across a range of “problematic” genomic locations can be overcome. In this way, the foundation for discovering disease related SVs would be laid. At the same time, short-read sequencing can help correct the higher error rate of long read sequencing on each individual nucleotide to facilitate better SNP and small indel calling. Finally, with the incorporation of multi-omics data/single-cell data, researchers are able to fill the gap between genotype and phenotype, therefore, uncovering the full landscape of disease etiology.

## Author contributions

W-PL and L-SW conceived the idea. HW and W-PL carried out the structure of the writing and contributed to manuscript writing. GS supervised the project. All authors contributed to the article and approved the submitted version.

## Funding

This work was supported by funding from the National Institute on Aging (RF1AG074328 and U24-AG041689) and University of Pennsylvania ADRC pilot project (R01-AG064877-02S1).

## Conflict of interest

The authors declare that the research was conducted in the absence of any commercial or financial relationships that could be construed as a potential conflict of interest.

## Publisher’s note

All claims expressed in this article are solely those of the authors and do not necessarily represent those of their affiliated organizations, or those of the publisher, the editors and the reviewers. Any product that may be evaluated in this article, or claim that may be made by its manufacturer, is not guaranteed or endorsed by the publisher.
